# *Streptococcus constellatus* Peritonitis and Subsequent Septic Shock following Intrauterine Device Removal

**DOI:** 10.1155/2019/6491617

**Published:** 2019-08-04

**Authors:** Joan Tymon-Rosario, Jessica M. Atrio, Hyun Ah Yoon, David Erlichman, Veronica Lerner

**Affiliations:** ^1^Department of Obstetrics & Gynecology and Women's Health, Albert Einstein College of Medicine, Montefiore Medical Center, Bronx, NY, USA; ^2^Division of Family Planning, Department of Obstetrics & Gynecology and Women's Health, Bronx, NY, USA; ^3^Department of Pathology, Albert Einstein College of Medicine, Montefiore Medical Center, Bronx, NY, USA; ^4^Department of Radiology, Albert Einstein College of Medicine, Montefiore Medical Center, Bronx, NY, USA; ^5^Division of Minimally Invasive Surgery, Department of Obstetrics & Gynecology and Women's Health, Bronx, NY, USA

## Abstract

**Background:**

Previous reports have described cases of abscess formation by* Streptococcus constellatus *involving the oral cavity, gastrointestinal tract, and septic thrombophlebitis of the right ovarian vein with subsequent bacteremia and septic shock. Ascending infection from the genital tract to the fallopian tubes resulting in peritonitis from* Streptococcus constellatus *is a rare clinical circumstance where there is minimal information in the literature to guide its diagnosis, management, and expected prognosis.

**Case:**

A 36-year-old G3P0111 developed a tubo-ovarian abscess two weeks after intrauterine device (IUD) removal and then rapidly decompensated with septic shock from peritonitis due to* Streptococcus constellatus *infection. The patient was also newly diagnosed with diabetes and in diabetic ketoacidosis (DKA) on presentation. She received broad-spectrum antibiotic coverage and required two exploratory surgical procedures to obtain source control. Two Interventional Radiology- (IR-) guided drainage procedures were subsequently performed to drain remaining fluid collections. Her recovery involved a prolonged ICU stay. On hospital day seventy-three, after receiving approximately 8 weeks of antibiotics and the above noted procedures the patient was discharged to a subacute rehabilitation facility.

**Conclusion:**

* Streptococcus constellatus *is a highly pathogenic organism once a systemic septic infection has become established that can cause an ascending genital tract infection resulting in tubo-ovarian abscess formation, peritonitis, and septic shock.

## 1. Introduction

Pelvic inflammatory disease (PID) comprises a spectrum of inflammatory disorders of the upper female genital tract as well as the surrounding organs. This includes any combination of endometritis, salpingitis, oophoritis, peritonitis, perihepatitis, and/or tubo-ovarian abscess [1]. Recent studies have demonstrated that the proportion of PID cases that are attributable to* Neisseria gonorrhoeae or Chlamydia trachomatis *are declining, as <50% of women who are diagnosed with PID are found to be positive for either of these organisms [1–4]. The various microorganisms that comprise the vaginal flora such as the anaerobes, enteric Gram-negative rods,* Gardnerella vaginalis, Haemophilus influenza, Mycoplasma hominis, Ureaplasma urealyticum, Mycoplasma genitalium, *and* Streptococcus agalactiae *have been associated with PID [5, 6].

*Streptococcus constellatus* collectively with* Streptococcus anginosus* and* Streptococcus intermedius* constitute the* Streptococcus anginosus* (formerly* Streptococcus milleri*) group [7].* Streptococcus constellatus* is a commensal organism of the human oral cavity, urogenital region, and intestinal tract. The distinguishing feature of this organism is its ability to form abscesses. In rare cases these abscesses can spread hematogenously and result in bacteremia with metastatic abscesses in other organs, subsequent septic emboli, septic thrombophlebitis, and septic shock as a rare complication. Previous reports in the literature have described such cases of abscess formation involving the oral cavity, upper gastrointestinal tract, and even septic thrombophlebitis of the right ovarian vein that have resulted in bacteremia and septic shock [7, 8].

Ascending infection from the genital tract to the fallopian tubes resulting in peritonitis in previously healthy women after intrauterine device (IUD) placement has been described in the literature; however, there is general consensus that this incidence is very low with PID occurring in 0.1-0.5 percent of patients after IUD placement [9, 10]. The causative pathogenic species that have been associated with serious infections with IUD use include* Staphylococcus, Streptococcus, *and* Actinomyces *species [9, 11–17]. We report a unique clinical circumstance in which* Streptococcus constellatus *caused an aggressive ascending genital infection with an unusual clinical course after IUD removal.

## 2. Case Description/Summary

**Figure 1:**
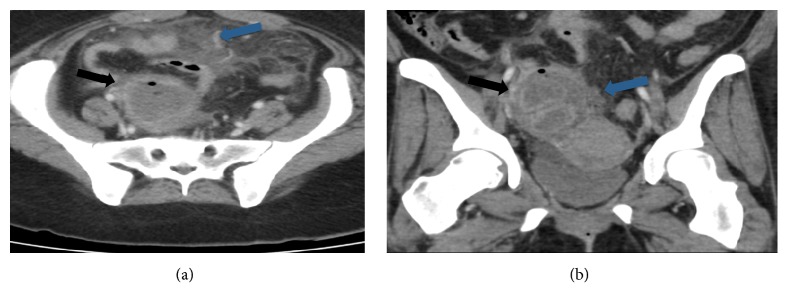
Patient's computed tomography (CT) scan of the abdomen and pelvis before surgery.  Figure 1(a) Transverse view.  Figure 1(b) Coronal view. Black arrow demonstrates right adnexal peripherally enhancing mass with extensive surrounding inflamatory changes and foci of air compatible with tubo-ovarian absess (TOA). Blue arrow demonstrates extensive inflitration of teh mesentery with inflamatory changes as well as fluid concerning for TOA rupture and seeding of the mesentary.

A 36-year-old G3P0111 with a past medical history significant for seizure disorder and morbid obesity (Body Mass Index 33 kg/m^2^) presented with a chief complaint of progressively worsening abdominopelvic pain, nausea, and vomiting for one day. Two weeks prior she presented to an outside facility for right-sided abdominopelvic pain and dyspareunia at which time her intrauterine device was found to be malpositioned with the IUD was low lying and predominantly in the cervix but otherwise there was no evidence of pelvic pathology on transvaginal ultrasound imaging. She subsequently had the intrauterine device removed by an outside gynecologist prior to her presentation to our facility. On presentation to the emergency room, she was found to be tachycardic but normotensive and afebrile. Her laboratory values were significant for white blood cell count of 18.4-cells/mm^3^ and a glucose level of 443 mg/dL. Computed tomography (CT) scan of the abdomen and pelvis demonstrated a right-sided tubo-ovarian abscess that was 8 cm in diameter with foci of internal air and extensive surrounding inflammatory changes (Figure 1).

The patient had not received primary care for a prolonged period of time. She was subsequently found to be in diabetic ketoacidosis with newly diagnosed type 2 diabetes mellitus and a hemoglobin A1c level of 14% requiring aggressive intravenous fluid hydration and insulin. She also had a low grade temperature so one set of blood cultures were obtained and she was initiated on empiric antibiotics with intravenous gentamicin and clindamycin pending complete work-up. Interventional radiology (IR) was consulted for possible drainage of the tubo-ovarian abscess but the location of the abscess was not amenable for a safe percutaneous approach secondary to bowel loops and major arteries being in close proximity to the abscess. While she initially had mild improvement of her abdominal pain with downtrending fever curve and leukocytosis, her clinical course was complicated by oliguria and acute renal insufficiency which was thought to be due to administration of gentamicin in the setting of intravascular volume depletion. In light of the patient's deteriorating renal function and reported IgE-mediated allergy to penicillin, the antibiotic regimen was changed to oral levofloxacin and metronidazole.

On hospital day three, the patient was noted to be somnolent with decreased mentation and labored breathing. Her capillary blood glucose level was 74 mg/dL and she had fevers up to 102.9°F with the initial blood culture detecting growth of* Streptococcus constellatus*. Critical care medicine and infectious diseases consultations were obtained for impending septic shock and the antimicrobial regimen was empirically broadened to vancomycin, meropenem, and micafungin. She subsequently underwent endotracheal intubation for airway protection and was initiated on vasopressor support and transferred to the intensive care unit (ICU). Consent for surgical source control was obtained from the health care proxy, who endorsed that the patient desires to preserve her fertility.

On hospital day four, the patient underwent laparoscopic lysis of adhesions, drainage, and debridement of a presumed right tubo-ovarian complex, partial right salpingectomy, and abdominal washout. Upon entry into the abdominal cavity there were copious purulent fluid and diffuse inflammatory changes throughout the abdomen and pelvis. Secondary to inflammation and friability of the tissue, the superior and medial portion of the right tubo-ovarian complex was resected with sufficient distance from the sidewall and the remaining small portion of the wall was left in situ. Of note, mostly blood clot was noted in the cavity of the complex, which was consistent with hemorrhagic cyst; however, extreme inflammation and friability of the complex made it difficult to clearly identify structures but no frank pus was noted within the adnexal structures. The uterus did not appear to be involved with any of these inflammatory processes. Postoperatively, the patient still had persistent high-grade fevers and continued to remain intubated on vasopressor support. On postoperative day two, a discussion was held with her health care proxy about her lack of clinical improvement and agreement for reoperation with possible hysterectomy. She was brought back to the operating room where she underwent an exploratory laparotomy, abdominal washout, lysis of adhesions, partial omentectomy, right salpingo-oophorectomy, right ureterolysis, and cholecystectomy in an attempt for further source control. During the procedure, multiple pockets of pus in the middle and upper abdomen between loops of bowel and omentum were noted and drained, with extensive fibrinous exudate noted to be overlying the small bowel, large bowel, and pelvis. Right salpingo-oophorectomy was performed to assure that no potential source for infection was being left behind but no frank pockets of pus were noted at this time which were similar to her initial intraoperative findings. The gallbladder was noted to be gangrenous consistent with acalculous gangrenous cholecystitis, which prompted cholecystectomy to be performed. Drains were placed and the fascia was closed, but the incision was left open to heal via secondary intention.

**Figure 2:**
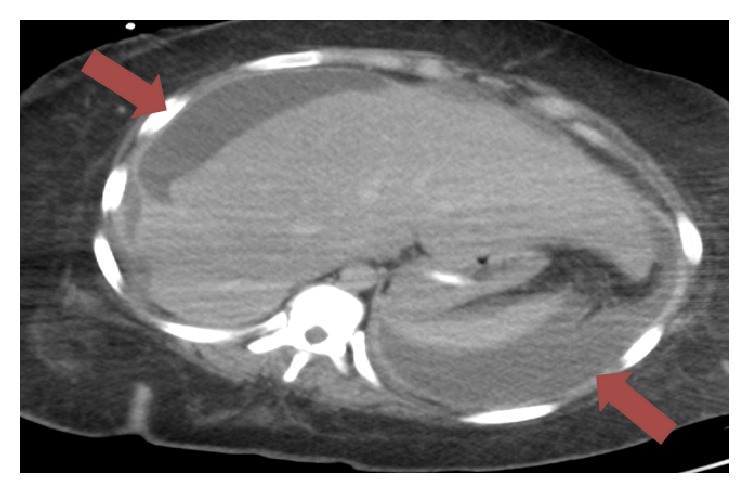
Patient's computed tomography (CT) scan of the abdomen and pelvis after her two surgeries looking for collections that remained. Red arrows demonstrate complex perihepatic and perisplenic ascites with peritoneal enhancement concerning for postoperative purulent collections. The collections were subsequently drained percutaneously and found to represent abscesses.

After undergoing the above procedures, the patient remained intubated on vasopressor support with high-grade fevers and persistent leukocytosis. She slowly was weaned off of vasopressor support, had an improvement in her ventilator settings, and became more responsive. Overall, her postoperative course was complicated by anemia necessitating blood transfusions and a left-sided lower extremity deep venous thrombosis with a right subsegmental pulmonary embolus requiring therapeutic anticoagulation. Transthoracic echocardiography did not reveal any valvular vegetations. The patient also developed a sacral decubitus ulcer as a result of her physical deconditioning. In addition, a wound vacuum was also placed over her abdominal incision secondary to poor wound healing. A peritoneal fluid culture from her initial procedure had detectable growth of* Streptococcus constellatus *that was susceptible to penicillin and ceftriaxone identical to the organism from the initial blood culture. The final pathology from her initial procedure demonstrated a hemorrhagic cyst with necrotic tissue and blood clot. The final pathology from her second procedure demonstrated right ovarian tissue with a hemorrhagic corpus luteum, fallopian tube, and fibroadipose tissue showing acute suppurative and chronic inflammation. It also demonstrated the gallbladder had acute suppurative and chronic inflammation, predominantly seen in the serosal surface. Another CT scan of the abdomen and pelvis was performed demonstrating bilateral upper quadrant fluid collections (Figure 2). Successful IR-guided drainage of the upper abdominal fluid collections was performed with drain placement. She was maintained on broad-spectrum antibiotics coverage with vancomycin and meropenem for approximately 2 weeks given persistent high grade fevers with high pressor requirements and a high concern for an uncontrolled source. As repeated blood cultures and repeated abdominal drainage cultures did not detect any bacteria so the regimen was deescalated to ceftriaxone and metronidazole.

It was also discussed that given the patients poor respiratory status with ventilation settings of both high fraction of inspired oxygen (Fi02) and positive end-expiratory pressure (PEEP) requirements that if further surgical intervention was indicated it would likely require use of extracorporeal membrane oxygenation (ECMO). Further surgery would also put her at high risk of a surgical complication such as enterotomy. Moreover, additional surgical intervention was deferred as the likely source of infection at this time was abdominal and successful IR-guided drainage with peritoneal fluid cultures demonstrated no growth of any organisms.

On postoperative day 16 after her second procedure, the patient remained ventilator dependent and thus underwent tracheostomy placement with concurrent abdominal wound debridement and replacement of the wound vacuum. Vaginal cultures were also obtained at the time of this procedure and demonstrated* Candida albicans *for which the patient received antifungal treatment. She then subsequently had a percutaneous endoscopic gastrostomy (PEG) tube placed but it failed so she remained on nasogastric tube feeds. Despite continued treatment with broad-spectrum IV antibiotics, the patient still had persistent fevers and thus the decision was made to perform another CT chest, abdomen, and pelvis to evaluate for any further intra-abdominal collections or alternative source of infection. Imaging demonstrated a significant decrease in size of the prior collections and concern for pneumonia. The patient's antibiotic regimen was adjusted per the infectious disease consultation recommendations. The intraperitoneal drains were removed as they had minimal output and could pose as a potential nidus for infection. The patient still remained intermittently febrile and thus on postoperative day 30 from her exploratory laparotomy she obtained another CT chest, abdomen, and pelvis which demonstrated an interval decrease in small bilateral pleural effusions and bibasilar atelectasis/consolidation but with upper abdominal collections again present. IR-guided drainage was again successfully performed after which the patient defervesced. The patient was afebrile after postoperative day 36 from her second procedure, without a leukocytosis and she was able to converse coherently with a tracheostomy collar in situ. Her upper abdominal drain was removed and she completed additional two weeks of ceftriaxone and metronidazole from time of last abdominal drainage. In total, she had received antibiotic coverage for approximately 8 weeks during her complicated clinical course. On hospital day seventy-three, the patient was discharged in stable condition to a subacute rehabilitation facility.

## 3. Discussion

This case presents a rare clinical scenario wherein a patient developed a tubo-ovarian abscess two weeks after intrauterine device (IUD) removal and then rapidly decompensated with resulting peritonitis and septic shock from the organism* Streptococcus constellatus *with a prolonged hospitalization due to metastatic spread of the infection. The spectrum of inflammatory disorders of the upper female genital tract typically results from the infection of various microorganisms that comprise the vaginal flora. Although* Streptococcus constellatus *is a commensal of the human oral cavity, urogenital and intestinal tract, there have been rare case reports of its abscess-forming capabilities that led to bacteremia, septic shock, and potentially death. Haidar et al. presented a case of* Streptococcus constellatus *bacteremia associated with septic thrombophlebitis to the right ovarian vein extending into the inferior vena cava [7]. Our case adds new insight on the management and treatment of severe life-threatening complications resulting from the abscess formation by* Streptococcus constellatus *in the female genital tract with resulting peritonitis and septic shock [7, 8].

Ascending infection from the genital tract to the fallopian tubes resulting in peritonitis in previously healthy women after intrauterine device placement (IUD) has been described in the literature [9]. The causative pathogenic organism described in these case reports is* Streptococcus pneumoniae*.* Streptococcus constellatus *although well known for its abscess-forming capability potentially resulting in septic shock has not yet been described as a causative organism from ascending genital infection resulting in tubo-ovarian abscess formation with septic shock. Therefore, the severe pathologic potential of* Streptococcus *organisms and their role in pelvic inflammatory disease is an area of research that warrants further investigation. Additionally, although one cannot make an exact causal relationship between the patient's IUD removal and subsequent infection, ascending genital infection after IUD removal rather than only after placement is also an area of investigation, which should be further explored.

The care of this patient exemplifies the importance of a multidisciplinary team approach in the management of a medically complex patient with an unusual disease process. Given the patient's tenuous clinical course, a multidisciplinary team conference was held to discuss her treatment plan. During the conference, it was postulated that* Streptococcus constellatus*, a commensal of the human genitourinary and gastrointestinal tract, may have had spread due to mucosal injury in the setting of the intrauterine device malposition and/or removal that was complicated by hematogenous spread to the fallopian tube, intraperitoneal cavity, and the gastrointestinal tract causing likely polymicrobial abscesses. In addition, new diagnosis of diabetes mellitus complicated by diabetic ketoacidosis likely contributed to dysfunctional host defense against this pathogen allowing aggressive spread. This theory was consistent with the fact that the final pathology was more consistent with serositis and there was no purulent material noted along the pelvic organs on the second case after the partial tubo-ovarian abscess. Additionally,* Streptococcus constellatus *is known to create extensive local abscesses and to cause septic emboli with a high morbidity and mortality rate [7, 8].

We acknowledge that a shortcoming to our findings is that this is a single case report of a personalized treatment plan, which limits the application of our findings to other patients. However, this case highlights the morbidity and potential mortality that can result from a pelvic infection with* Streptococcus constellatus *causing peritonitis and septic shock. Therefore, care for those patients with such a pelvic infection caused by* Streptococcus constellatus *likely will require not only medical management with broad-spectrum antibiotics as the abscesses are often polymicrobial with other anaerobic organisms and gram negative pathogens but also early surgical intervention(s) for complete source control. The various treatment options and the management of the possible complications of* Streptococcus constellatus *infection require further assessment in order to improve future patient care.

## Additional Points

(1)* Streptococcus constellatus *can cause rapidly progressing and life-threatening ascending genital tract infections that can cause abscess formation, peritonitis, and septic shock. (2) Rapid clinical deterioration in a patient diagnosed with a tubo-ovarian abscess must prompt one to consider infection with unconventional microorganisms such as* Streptococcus constellatus*. (3) Peritonitis and septic shock may complicate the medical management of a tubo-ovarian abscess caused by* Streptococcus constellatus *necessitating early surgical intervention.
